# Supplementary material for: Transmission-Blocking Vaccines against Schistosomiasis Japonica
Source: Int J Mol Sci. 2024 Jan 30;25(3):1707. doi: 10.3390/ijms25031707 (PMC10855202; doi:10.3390/ijms25031707)
Supplement: Supplementary file 1 [file ijms-25-01707-s001.zip › ijms-2814849-supplementary.pdf]

Supplementary Table 1: The three common species of Schistosoma affecting humans

**Species**	***Schistosoma haematobium***	***Schistosoma mansoni***	***Schistosoma japonicum***
**Distribution**	Africa, Middle East, Corsica (France)	Africa, the Middle East, the Caribbean, Brazil, Venezuela, and Suriname	South of China, Philippines, and Indonesia
**Intermediate host (snails)**	*Bulinus* spp [24] 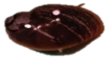	*Biomphalaria* spp [24] 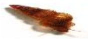	*Oncomelania* spp [24] 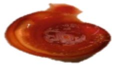
**Eggs**	Laid singly (150/day [24]) and excreted in urine. 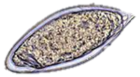 Terminal spine	Laid singly (300/day [24,25]) and excreted in faeces. 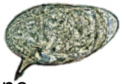 Lateral spine	Laid in clusters (1000-2200/day [24,25]) and excreted in faeces. 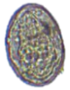 Small (almost invisible) lateral spine
**Definitive host (Human and mammalian animals)**	Mostly Human	Mostly Humans and rodents.	Humans and about 46 domestic animals [37] including water buffalo, cattle, sheep, dogs, baboons, pigs, and rodents.
**Host pathology**	Urogenital schistosomiasis results in haematuria, genital lesions, vaginal bleeding scarring, calcification, kidney failure, bladder squamous cell carcinoma, and infertility.	Hepato- intestinal schistosomiasis causes liver fibrosis, ascites, portal hypertension, hepato-splenomegaly, jaundice, CNS lesions.	
